# Perceived Needs Versus Predisposing/Enabling Characteristics in Relation to Internet Cancer Information Seeking Among the US and Chinese Public: Comparative Survey Research

**DOI:** 10.2196/24733

**Published:** 2021-01-11

**Authors:** Di Zhang, Hongchao Hu, Zhen Shi, Biao Li

**Affiliations:** 1 School of Journalism and Communication Renmin University of China Beijing China

**Keywords:** HINTS, health information seeking behavior (HISB), China, United States, comparative research, cultural sensitivity

## Abstract

**Background:**

Since the rise of the internet, online health information seeking has become a worldwide phenomenon. However, health and health communication are inherently culture bound. A data-driven cross-country comparison enables us to better understand how cultural factors moderate the association between individual-level determinants and online health information seeking.

**Objective:**

The objective of the study was to examine similarities and differences in determinants of internet cancer information seeking between the US and Chinese general public (excluding cancer patients and survivors) under the framework of a behavioral model of health services use.

**Methods:**

This study used Health Information National Trends Survey (HINTS) 2017 (US data) and HINTS-China 2017 data to answer the research question. It focused on people with no cancer history and with internet access. For the HINTS 2017, the sample size was 2153; for the HINTS-China 2017, the sample size was 2358. To compare China and the United States, the researchers selected the same set of study variables for each dataset. Under the framework of the behavioral model of health services use, these predictors were predisposing factors, enabling factors, and need factors.

**Results:**

In terms of the predisposing factors, a higher age, college degree or above, being currently unemployed, and having a family history of cancer were associated with internet cancer information seeking for the Chinese respondents; none of these factors were related to information seeking for the US respondents, although a lower age was associated with information seeking. Regarding the enabling conditions, lower trust in family members and friends as reliable information sources was the only factor associated with information seeking for the Chinese respondents, while no enabling factor was related to information seeking for the US respondents. Regarding the need factors, perceived health status was not related to information seeking for the Chinese respondents, while perception of poorer health condition was related to information seeking for the US respondents. Higher cancer fear was related to information seeking for both groups, but the magnitude of association was smaller for the Chinese respondents than for the US respondents.

**Conclusions:**

Overall, under the framework of the behavioral model of health services use, the results based on multivariate logistic regression reveal clear patterns of cross-country/cultural differences in the factors associated with internet cancer information seeking behaviors: predisposing characteristics and enabling conditions are more important in China, while perceived needs are more significant in the US. Such differences might reflect possible US-China differences in job environment (eg, job pressure) and culture (individualism vs collectivism and family structure).

## Introduction

### Background

A milestone in monitoring the US public’s access to and use of health information is the Health Information National Trends Survey (HINTS) initiated by the National Cancer Institute [1], which provides valuable guidance for practitioners. With the cultural sensitivity approach becoming increasingly popular [2], calls for the expansion of the HINTS research programs into other parts of the world have been made, and HINTS-China pioneered this international expansion [3]. The pilot international HINTS survey has been conducted twice in China, in 2012 and 2017 [3]. The replication of the HINTS research program in countries and regions outside the US based on similar measurement schemes benefits global health communication research in numerous ways; one is that it allows scholars to compare health information seeking behaviors among countries [3]. Data-driven, multicountry comparisons enable us to better understand how cultural factors moderate the association between individual-level determinants and health information seeking, which further helps practitioners evaluate the probability of successful health communication intervention designs in one country being transferable to other countries [3].

To fulfill the mission of the international expansion of the HINTS program, this study used HINTS and HINTS-China data collected in 2017 [4] to examine similarities and differences in determinants of online cancer information seeking between the US and Chinese general public (excluding cancer patients and survivors) under the framework of the behavioral model of health services use [5,6]. Since the rise of the internet, online health information seeking has attracted worldwide scholarly attention. However, most scholars have conducted such studies in a single country. Some scholars have designed comparative studies across countries [7,8], but they have normally been based on small nonprobability college student samples. Therefore, the results of this study can contribute to the health communication literature by generating more reliable insights into country differences in health information acquisition, such as internet cancer information seeking.

### Literature Review

#### Determinants of Online Health Information Seeking

We not only reviewed studies on cancer information seeking on the internet, which are limited in number, but also reviewed research concerning generalized online health information seeking to present a more complete picture of the factors that potentially play a role. Regardless of the theoretical models used, the predictors of major concern in existing studies include mainly demographics [9], structural characteristics [9], and perception variables [10]. Additionally, such studies often assess physiological indicators, but they have rarely been found to be statistically significant [11].

The effects of demographic and structural variables are rather stable. According to HINTS data, Americans who refer to the internet for health information are younger than those who do not [9,11]. Additionally, age moderates the positive association between trust in internet information and online health information seeking [11]. Previous research has generally found that higher socioeconomic status is associated with greater health information acquisition [9,12,13]. Married people reported a higher frequency of internet use to search for health information [12]. However, the results related to gender are mixed. Some studies have found that women are more likely to use the internet to obtain health information than men [12,14], while others have found that gender does not make a difference [9,15].

Most studies on generic health information seeking concentrate on people’s perceptions of risk, personal health status, self-efficacy, media trust, social support, and satisfaction with caregivers. Those with higher levels of perceived risks and fears and lower levels of confidence about their health status are more likely to search for health information on the internet [10,16-18]. Those with higher internet self-efficacy are more likely to obtain health information online [10,19-21]. Those who trust the internet as an accurate and reliable information source or channel are more likely to rely on it to obtain medical information [11,18,19]. Additionally, trust in other players or information sources is positively associated with online health information seeking behaviors. Trust in health information from family members, for instance, is positively related to internet health information seeking behaviors among the US population [22]. Social support is positively associated with web searches for health information [21,22]. People with more social ties can seek health information from their social ties when finding it difficult to obtain online [23]. In a similar vein, patients who are unsatisfied with doctors tend to use the internet for medical information [24].

#### Behavioral Model of Health Services Use and Health Information Seeking Predictors

We used the behavioral model of health services as the theoretical framework to organize the predictor variables of internet cancer information seeking, as the model evaluates the extent to which 3 sets of predictor variables (predisposing factors, enabling factors, and need factors) influence people’s use of health services [5,6,25]. The model also suggests a causal order: predisposing characteristics such as demographics and social structures are exogenous; enabling factors are necessary conditions for people to use medical services; and need variables (perceptions of needs) must be defined for use to take place [25]. According to the model, the 3 categories of factors encompass both contextual and individual characteristics [26]. Contextual characteristics refer to the environment and circumstances of health care service access, health care provider characteristics, and community characteristics, which are often measured at the aggregate level instead of the individual level [26]. Individual characteristics include demographics, social factors, personal beliefs, personal financial status, personal perceptions of health status, and objective evaluation of one’s physical status [26]. Contextual characteristics may influence access to health care services through individual characteristics [26]. Although the behavioral model of health services was originally proposed to explain people’s use of health services, scholars have successfully applied it to explain variations in health information seeking or channel use [27,28]. Additionally, the model incorporating both contextual and individual characteristics is suitable for the comparative nature of this study because the US and China differ in terms of community structure, culture, and health traditions [29-32].

This study focused on how individual-level predisposing, enabling, and need factors are associated with information seeking and how their relationships vary as a function of the country group. Predisposing characteristics were age; gender; education; ethnicity; the status of a person in his or her community; general health attitudes, values, and knowledge; and genetic characteristics [25,26]. Enabling resources were access to health facilities, access to medical personnel, and access to the medical system, including health care policies, personal income, health insurance, social relationships, and organizational factors [25,26]. Need factors were an individual’s evaluation of his or her health status, need for medical services, and concerns about health problems [25,26]. With reference to a study that analyzed HINTS-China 2012 data [27], the researchers of this study selected age, gender, educational attainment, marital status, and BMI as predisposing factors. Enabling factors were current occupational status, household income, and trust in social institutions and information channels. Need characteristics were perceived health status, fatalism about cancer, fear of cancer, and perceived cancer risks. Although these variables were measured at the individual level, Anderson’s model suggests that contextual characteristics can exert an influence through individual-level characteristics, which can reflect meso- or macrolevel contextual influences [29-32]. For instance, occupational status and household income can reflect the influence of the mesolevel working environment; trust in family members as health information sources can reflect mesolevel family influences; and perceived need factors can reflect macrolevel cultural influences.

#### Differences Between the US and China

Health communication scholars have paid increasing attention to internet health information seeking in the US and Europe, but little is known about China, where more than 4 million patients were diagnosed with cancer in 2015 [33]. Health and health communication are inherently culture bound [2]. Research findings generated from the West may not be readily applicable to countries such as China and Japan, which differ significantly from the US and other Western countries. Because this study compares China and the US, the following sections focus on cultural and structural differences between the two countries.

China and the US vary in issues related to cancer control. First, the types of common cancers in China differ from those in the US. In China, lung cancer has the highest incidence rate [34]. In the US, prostate cancer has the highest incidence rates for men and breast cancer for women [35]. Second, the Chinese medical care system is not as well established as the US medical care system [36]. With market-oriented reforms, the traditional system of the government and state-owned enterprises taking care of all medical needs has collapsed, while the emerging medical insurance system is far from satisfactory [36,37]. Third, many Chinese people believe in Chinese traditional medicine based on a holistic worldview [32]. Some Chinese cancer patients choose integrated therapies combining Western and Chinese traditional medicines [38]. Fourth, the doctor-patient relationship in China has been deteriorating for more than a decade [39]. Unlike their US counterparts, Chinese families do not have family doctors and, as such, visit comprehensive and specialized public hospitals even for minor conditions such as colds and coughs; as a result, a huge workload makes it difficult for doctors and nurses in China to find time to communicate with patients and their family members [39]. Workplace violence involving doctors and patients/family members has often made Chinese media headlines [40]. The commercialized medical care system in China has further eroded public trust in doctors, who people believe conspire with pharmaceutical companies to make money off of patients [41].

Chinese culture differs from US culture. China is collectivistic, while the US is more individualistic [30]. Individualism versus collectivism is an important construct in health psychology; individuals in individualistic cultures tend to emphasize personal wellbeing [42]. Individualism versus collectivism moderates the relationship between negative affect and psychological wellbeing, and the strength of the negative association between negative affect and wellbeing is stronger in individualistic cultures than in collectivistic cultures [43]. Additionally, Chinese people are commonly believed to be more family oriented than their US counterparts [44]. In terms of social structure, although the one-child policy has destroyed the traditional Chinese extended family structure, families remain central in Chinese life [45]. Family members remain close to each other by staying in a so-called networked family, even though it is no longer characterized by physical proximity among members [45]. Comparing differences in the strength of family-related variables can partly address criticism that the behavioral model of health services use lacks consideration of social relationships [25].

Although the US has a longer history of internet commercialization, China has recently begun to take the lead in the commercialization of new media applications, which have penetrated every social stratum [46]. A case in point is that most street beggars print out QR codes to collect donations in the street. Therefore, Chinese people increasingly use various types of internet applications to obtain health information [47]. Additionally, China is ahead of the US in the delivery of internet medical services because venture capitalists and entrepreneurs have invested heavily in the internet medical care industry [48]. It is common for Chinese people to make medical appointments with doctors using internet applications such as hospital portals, social media, and mobile applications [47].

On the basis of the preceding discussion, this study plans to answer the following research question:

How do China and the US differ in the associations between predisposing/enabling/need factors and internet cancer information seeking behaviors among people without a history of cancer?

## Methods

### Data Sources

This study used HINTS 2017 (US data) and HINTS-China 2017 data to answer the research question. HINTS 2017 had a final sample size of 3285. Of the respondents, 2756 had no history of cancer (current patients or survivors); thus, these respondents were the focus of the study.

HINTS-China is an effort jointly launched by the Chinese Ministry of Health Center for Health Education, Renmin University of China, the Chinese National Cancer Center, and George Mason University and was recently joined by Beijing Normal University [27]. HINTS-China modeled the majority of the question items in HINTS and was adapted to local Chinese characteristics and the Chinese population. In the pilot phase, the Chinese survey was conducted twice, in 2012 and 2017, and was administered only in the Chinese capital city, Beijing, and Anhui Province’s capital city, Hefei. Beijing was chosen because it is a megacity and the economic center of China, and Hefei was selected because it is a less developed, second-tier Chinese city that represents a more typical Chinese city. HINTS-China 2017 followed the multistage stratified random sampling strategy of HINTS-China 2012 [25]. HINTS-China 2017 was conducted using in-person interviews, had a valid response rate of 64%, and resulted in a sample size of 3090, of whom 10 had a history of cancer.

Because online health information seeking is directly related to physical access to the internet, this study excluded those without internet access from the final model because it is obvious that those without internet access cannot search for cancer information on the web. The US sample contained 750 respondents who had never accessed the internet, and the Chinese sample had 723. Thus, the sample sizes of cancer-free populations with internet access were 2,153 and 2,358 for the US and China, respectively.

To compare China and the US, the researchers selected the same set of study variables as predictors in HINTS 2017 and HINTS-China 2017. Additionally, there were slight differences in the measurement scales of these predictors between countries, so recoding was conducted to facilitate comparison. Measurements of the predisposing, enabling, need, and outcome variables were as follows.

### Variable Measurement

#### Predisposing Variables

Gender was recoded as female, with 1 = female and 0 = male. Educational attainment for the US and China was converted from 7-point and 6-point scales, respectively, to 4-point scales, with 1 = “Less than high school,” 2 = “High school,” 3 = “Vocational school,” and 4 = “College and above.” Marital status for both countries was recoded as “Currently married,” with 1 = “Yes” and 0 = “No.” Similarly, current occupational status was converted to “Currently employed,” with 1= “Yes” and 0 = “No.” Current smoking status was assessed by 2 items, which were the same in both countries’ surveys. The first item was a filter question, “Have you smoked at least 100 cigarettes in your entire life?,” with 2 choices, (1) “Yes” and (2) “No.” Only those who answered “Yes” were presented with the second item, “How often do you now smoke cigarettes?,” with 3 choices (1) “Every day,” (2) “Some days,” and (3) “Not at all.” To combine the items into a single item, the researchers counted “No” for the first item as “Not at all” for the second item. BMI was calculated from height and weight using the standard formula. Both the Chinese and US surveys collected height and weight data. Family cancer history was measured using one item in both surveys. The US survey had 3 choices, (1) “Yes,” (2) “No,” and (3) “Not sure.” The Chinese version broke “Yes” into “Close relatives” and “Distant relatives.” They were recoded as a single variable, with 1 = “Yes” (including “Close relatives” and “Distant relatives”) and 0 = “No or not sure.”

#### Enabling Variables

Household annual income was originally measured at the interval level in China, which differs from the categorical income range used in HINTS 2017. Income in the Chinese version was recoded as a categorical variable in 4 quartiles (from “50,000 RMB yuan and below” to “150,001 RMB yuan and above” with a 50,000-RMB interval). Income in the US version, with 9 original categories, was also recoded in quartiles: “$0 to $19,999,” “$20,000 to $49,999,” “$50,000 to $99,999,” and “$100,000 or more.”

HINTS in both countries measured people’s trust in social institutions and information channels as reliable health information sources. However, the Chinese and US surveys differed slightly in the design of the attributes of each question item. First, the US survey measured only people’s trust in the generic internet, while the Chinese survey measured people’s trust in 8 typical internet applications. Then, the researchers averaged the trust score for the 8 internet applications to create an overall score for trust in the internet for the Chinese data. Second, the US survey used a single item to measure people’s trust in family or friends, while the Chinese survey measured them separately. Thus, the researchers averaged them to create a single score. Third, the US survey measured people’s trust in newspapers or magazines, while the Chinese survey measured them separately. The researchers created a new item using the mean score. Fourth, the US survey measured trust using a 4-point scale, with 1 = “Not at all” and 4 = “A lot.” In contrast, the Chinese survey measured trust using a 5-point scale, with 1 = “Very untrustworthy” and 5 = “Very trustworthy.” The 4-point scale used in the US survey was converted numerically into a 5-point scale to make the comparison more straightforward. Taking into account the goal of the study, the researchers categorized trust variables into 5 groups. The first category was trust in social institutions, including government agencies, religious organizations, and charities, which were combined into an additive index. The second category was trust in traditional media channels, including print media, television, and radio, which were combined into an additive index (Cronbach alpha for US=.77; Cronbach alpha for China=.85). Trust in the internet, doctors, and family members and friends were used as they were in the following logistic regression analysis.

#### Need Variables

Self-confidence about personal health or perceived health status was measured on a 5-point scale, with 1 = Poor and 5 = Excellent. The scales for both countries were the same.

Both surveys measured fatalism about cancer, which was assessed by 4 items such as “It seems like everything causes cancer” and “When I think about cancer, I automatically think about death.” The US survey used a 4-point scale, with 1 = “Strongly agree” and 4 = “Strongly disagree.” The scale in the Chinese survey was slightly different, with 1 = “Strongly disagree” and 5 = “Strongly agree.” The scale in the US survey was reversed and numerically converted into a 5-point scale, thus allowing the researchers to more easily compare the magnitudes of the coefficients. They were added to an index (Cronbach alpha for US=.63; Cronbach alpha for China=.75).

Fear of cancer was assessed using a single item on a 5-point scale, with 1= “Not at all” and 5= “Extremely.” The surveys for the two countries were identical.

Perceived cancer risk was assessed by asking the respondents, “How likely are you to get cancer in your lifetime?” with 1 = “Very unlikely” and 5 = “Very likely.” The surveys for the two countries were identical.

#### Outcome Variable

Online cancer information seeking was the outcome variable. In HINTS 2017, online cancer information seeking was binary, with 1 = “Yes” and 0 = “No,” while HINTS-China 2017 measured it using a 4-point scale. To make them comparable, the researchers recoded “Never” and “Rarely” as “No,” and converted “Often” and “Sometimes” to “Yes.”

### Data Analysis

In the first step, the researchers presented descriptive statistics of all the variables under examination. In the second step, the researchers used multivariate logistic regression to explore US-China differences in the associations between the 3 categories of the predictor variables and internet cancer information seeking (Table 1). The first model of the multivariate analysis examined only the main effects of the predictors; odds ratios, which are exponentiated log odds, were presented. The second model incorporated interaction terms of the country group (the US vs China) and predictor variables to examine between-country variation. The odds ratios of interaction terms are ratios of odds ratios. In the third step, the researchers presented conditional odds ratios of each predictor variable for each country group separately, and those predictor variables with statistically significant interaction terms were the focus of the discussion.

The US HINTS sample had missing values in most variables selected for comparison, and the listwise approach would have resulted in the loss of 569 observations (over 25% of the US sample cases). To maximize the number of observations used in the analysis, this study used the R package MICE (Multivariate Imputation via Chained Equations) to impute the missing values. Specifically, the researchers applied the CART (classification and regression trees) algorithm for categorical variables and PMM (predictive mean matching) for numerical variables [49]. A total of 50 sets of data with imputed values were generated for the US and China, and the coefficients and standard errors of the logistic regression results for the 50 imputed datasets were pooled together using the Rubin rules [50].

**Table 1:** Odds ratio of logistic regressions of predisposing, enabling, and need variables of internet cancer information seeking.

Variables			Model 1 odds ratio (95% CI)		Model 2 odds ratio (95% CI)		
**Predisposing variables**							
	Female (vs male)		1.07 (0.89-1.28)		1.11 (0.83-1.49)		
	Age		0.99 (0.99-1.00)*		1.02 (1.00-1.03)*		
	**Education (vs less than high school)**						
		High school		0.88 (0.62-1.24)	1.08 (0.72-1.62)		
		Vocational school		1.23 (0.87-1.73)	1.58 (1.03-2.42)		
		College and above		1.53 (1.08-2.18)*	2.35 (1.50-3.68)*		
	Currently married (vs not)		1.15 (0.95-1.39)		0.97 (0.69-1.36)		
	Currently employed (vs not)		0.80 (0.66-0.98)*		0.66 (0.50-0.89)*		
	BMI		0.99 (0.97-1.01)		0.98 (0.94-1.03)		
	Family cancer history (vs not)		1.51 (1.25-1.82)*		2.23 (1.73-2.88)*		
	**Smoking status (vs every day)**						
		Some days		1.04 (0.61-1.78)	0.54 (0.20-1.46)		
		Not at all		1.12 (0.85-1.48)	0.94 (0.64-1.39)		
**Enabling variables**							
	**Annual family income (vs $0 to $19,999/50,000 RMB and below)**						

		$20,000 to $49,999 (50,001 to 100,000 RMB)		0.74 (0.58-0.94)*	0.70 (0.51-0.96)*		
		$50,000 to $99,999 (100,001 to 150,000 RMB)		0.65 (0.49-0.86)*	0.65 (0.43-0.99)*		
		$100,000 or more (150,001 RMB and above)		0.76 (0.57-1.02)	0.71 (0.48-1.06)		
	**Trust in information sources**						
		Social institutions		0.99 (0.95-1.04)	0.97 (0.90-1.04)		
		Traditional media		1.02 (0.98-1.07)	1.01 (0.95-1.08)		
		Internet		1.18 (1.05-1.32)*	1.43 (1.13-1.80)*		
		Doctors		0.93 (0.84-1.04)	0.96 (0.81-1.13)		
		Family and friends		0.87 (0.78-0.97)*	0.71 (0.59-0.86)*		
**Need variables**							
	Perceived health status		0.83 (0.75-0.93)*		0.94 (0.79-1.11)		
	Cancer fatalism		1.02 (0.99-1.05)		1.03 (0.99-1.08)		
	Cancer risk		1.02 (0.92-1.14)		0.97 (0.82-1.16)		
	Cancer fear		1.45 (1.33-1.58)*		1.28 (1.12-1.47)*		
	US (vs China)		0.78 (0.58-1.06)		2.03 (0.22-18.84)		
**Interaction terms**							
	US X Female		N/A^a^		0.96 (0.66-1.41)		
	US X Age		N/A		0.97 (0.95-0.99)*		
	US X High school		N/A		0.49 (0.21-1.11)		
	US X Vocational school		N/A		0.54 (0.24-1.18)		
	US X College and above		N/A		0.40 (0.18-0.90)*		
	US X Currently married		N/A		1.19 (0.77-1.82)		
	US X Currently employed		N/A		1.43 (0.95-2.14)		
	US X BMI		N/A		1.00 (0.96-1.05)		
	US X Some days		N/A		2.98 (0.87-1.16)		
	US X Not at all		N/A		1.57 (0.87-2.85)		
	US X Family cancer history		N/A		0.42 (0.29-0.61)*		
	US X $20,000 to $49,999 (50,001 to 100,000 RMB)		N/A		1.15 (0.68-1.93)		
	US X $50,000 to $99,999 (100,001 RMB to 150,000 RMB)		N/A		1.09 (0.59-1.99)		
	US X $100,000 or more (150,001 RMB and above)		N/A		1.17 (0.63-2.18)		
	US X Social institutions		N/A		1.03 (0.94-1.13)		
	US X Traditional media		N/A		1.00 (0.92-1.09)		
	US X Internet		N/A		0.80 (0.61-1.04)		
	US X Doctors		N/A		0.99 (0.79-1.25)		
	US X Family and friends		N/A		1.36 (1.08-1.73)*		
	US X Perceived health status		N/A		0.81 (0.65-1.00)*		
	US X Cancer fatalism		N/A		0.96 (0.90-1.02)		
	US X Cancer risk		N/A		1.16 (0.92-1.45)		
	US X Cancer fear		N/A		1.24 (1.03-1.49)*		

^a^N/A: Not applicable.

*Asterisks represent the coefficients that are statistically significant at the *P*=.05 level.

## Results

### Descriptive Statistics

As seen in Table 2, the HINTS 2017 and HINTS-China 2017 had roughly the same percentage of cancer-free male and female respondents. However, the US sample had a much higher mean age, greater educational attainment, a lower rate of being currently married, and fewer employed people than the Chinese sample. The average BMI for the Chinese sample was lower than that of the US sample, and the percentage of regular smokers in the Chinese sample was slightly higher.

Regarding the other selected variables, the Chinese sample had higher perceived health status, while the US sample had slightly higher cancer risk perception and fear of cancer. The cancer fatalism scores for both did not differ much. The US respondents reported many more relatives diagnosed with cancers than their Chinese counterparts. The US respondents reported higher trust in social institutions, the internet, and doctors, while the Chinese respondents had more trust in family members/friends and traditional media.

**Table 2:** Descriptive statistics of dependent and independent variables for cancer-free respondents of the Health Information National Trends Survey (HINTS) 2017 and the HINTS-China 2017.

Variables		Categories or scales	US (N=2756)	China (N=3080)
Online cancer information seeking		Yes, n (%)	411 (14.90%)	322 (11.70%)
**Predisposing variables**				
	Female	Yes, n (%)	1607 (58.30%)	1686 (61.17%)
	Age	Years, mean (SD)	54.4 (16.1)	35.0 (11.5)
	Education	Less than high school, n (%)	190 (6.90%)	494 (17.92%)
		High school, n (%)	507 (18.40%)	744 (27.01%)
		Vocational school, n (%)	813 (29.50%)	719 (26.10%)
		College and above, n (%)	1246 (45.20%)	798 (28.96%)
	Currently married	Yes, n (%)	1428 (51.80%)	1944 (70.55%)
	Currently employed	Yes, n (%)	1499 (54.40%)	2065 (74.94%)
	BMI	kg/m^2^, mean (SD)	28.45 (6.46)	22.6 (3.17)
	Smoking status	Every day, n (%)	282 (10.25%)	421 (15.26%)
		Some days, n (%)	104 (3.78%)	65 (2.37%)
		Not at all, n (%)	2370 (85.98%)	2270 (82.37%)
	Family cancer history	Yes, n (%)	1915 (69.47%)	620 (22.50%)
**Enabling variables**				
	Annual family income	$0 to $19,999 / 50,000 RMB and below, n (%)	511 (18.55%)	757 (27.47%)
		$20,000 to $49,999 / 100,001 RMB to 150,000 RMB, n (%)	752 (27.27%)	1081 (39.22%)
		$50,000 to $99,999 / 100,001 RMB to 150,000 RMB, n (%)	840 (30.47%)	400 (14.52%)
		$100,000 or more / 150,001 RMB and above, n (%)	653 (23.71%)	518 (18.79%)
	Trust in social institutions	3 items; 3-15, mean (SD)	8.72 (2.66)	8.49 (2.33)
	Trust in traditional media	3 items; 3-15, mean (SD)	7.54 (2.62)	8.78 (2.66)
	Trust in internet	1-5, mean (SD)	3.32 (1.07)	2.72 (0.75)
	Trust in doctors	1-5, mean (SD)	4.51 (0.83)	3.87 (0.96)
	Trust in family and friends	1-5, mean (SD)	3.10 (0.95)	3.85 (0.81)
**Need variables**				
	Perceived health status	1-5, mean (SD)	3.41 (0.95)	3.98 (0.78)
	Cancer fatalism	4 items; 4-20, mean (SD)	12.48 (3.34)	12.30 (3.19)
	Cancer risk	1-5, mean (SD)	3.06 (0.96)	2.24 (0.86)
	Cancer fear	1-5, mean (SD)	2.51 (1.10)	2.18 (1.01)

### Logistic Regression Analysis

Table 1 presents the results of logistic regressions of the factors of online cancer information seeking for both countries. Model 1 examined the main effects, and Model 2 analyzed the moderation by country group of the associations between the three sources of predictors and internet cancer information seeking.

The results of Model 1 suggest that younger people ﻿(OR=0.99, 95% CI 0.99-1.00) who had obtained at least a college degree ﻿(OR=1.53, 95% CI 1.08-2.18), were currently not employed (OR=0.80, 95% CI 0.66-0.98), and had a family history of cancer (OR=1.51, 95% CI 1.25-1.82) were more likely to search for cancer information on the internet. In terms of enabling conditions, those who earned a moderate income (OR=0.74, 95% CI 0.58-0.94; OR=0.65, 95% CI 0.49-0.86), trusted the internet as a reliable source of information (OR=1.18, 95% CI 1.05-1.32), and distrusted family members and friends as reliable information sources (OR=0.87, 95% CI 0.78-0.97) were more likely to search the internet for cancer information. In addition, those who perceived themselves to be in poor health (OR=0.83, 95% CI to 0.75-0.93) and feared cancer (OR=1.45, 95% CI 1.33-1.58) were more likely to search the internet for cancer information.

According to the results of the tests of interaction terms in Model 2, as shown in Table 1, the Chinese and US groups differed in the associations between predisposing, enabling, and need factors and internet cancer information seeking. Three predisposing factor interaction terms were statistically significant: age (OR=0.97, 95% CI 0.95-0.99), educational attainment (OR=0.40, 95% CI 0.18-0.90) and family history of cancer (OR=0.42, 95% CI 0.29-0.61). The coefficient of family history of cancer, the only enabling factor, differed across country groups (OR=1.36, 95% CI 1.08-1.73). In terms of need factors, perceived health status (OR=0.81, 95% CI 0.65 to 1.00]) and cancer fear varied (OR=1.24, 95% CI 1.03-1.49) between the Chinese and US sample groups.

On the basis of the significance tests of the interaction terms, the researchers further calculated conditional odds ratios of predictor variables for each country group (Table 3). The relationship of age with the outcome variable differed across the country groups: senior respondents in the US sample were less likely to seek cancer information on the web (OR=0.99, 95% CI 0.98-1.00), while the opposite was true for the Chinese sample (OR=1.02, 95% CI 1.00-1.03). For the US sample, education did not play a role, while Chinese respondents with degrees from vocational schools (OR=1.58, 95% CI 1.03-2.42) or colleges and universities (OR=2.35, 95% CI 1.50-3.68) were more likely to seek cancer information online than those who had not graduated from high school. The employment status of US respondents was not a statistically significant predictor (OR=0.95, 95% CI 0.71-1.25), while a working Chinese respondent was less likely to search for cancer information online (OR=0.66, 95% CI 0.50-0.89). Chinese respondents with close and distant relatives diagnosed with cancer were more likely to search for cancer information on the internet (OR=2.23, 95% CI 1.73-2.88), which did not apply to US respondents (OR=0.94, 95% CI 0.72-1.23).

According to Table 3, the odds ratio of trust in family and friends for the Chinese sample was negative and statistically significant (OR=0.71, 95% CI 0.59- 0.86), which did not hold true for the US sample (OR=0.97, 95% CI 0.84-1.11). Although the interaction terms between family annual income/trust in the internet and the outcome variable were not statistically significant, Table 3 suggests that the conditional odds ratios for the Chinese sample were statistically significant, while those for the US sample were not. While family annual income was not related to the outcome variable for the US sample, the Chinese respondents in the two income categories between 50,001 RMB and 150,000 RMB were less likely to use the internet for cancer cognition than those below 50,000 RMB (OR_50,001-100,000 RMB_=0.70, 95% CI 0.51-0.96; OR_100,001-150,000 RMB_=0.65, 95% CI 0.43-0.99). Additionally, the conditional odds ratio of trust in the internet for the Chinese respondents was statistically significant (OR=0.71, 95% CI 0.59-0.86), while that for the US respondents was not.

The odds ratios for two need variables were statistically significant. Perceived health status was associated with internet cancer information seeking only in the US sample (OR=0.76, 95% CI 0.65-0.87]), not for the Chinese sample (OR=.94, 95% CI 0.79-1.11). Cancer fear was related to the dependent variable for both samples. However, the conditional odds ratio for the US sample (OR=1.59, 95% CI 1.41-1.79) was larger than that for the Chinese sample (OR=1.28, 95% CI 1.12-1.47), and Table 1 suggests that the difference is statistically significant.

**Table 3:** Bivariate and conditional odds ratios (ORs) for logistic regression of predisposing, enabling, and need variables of internet cancer information seeking.

Variable			US		China	
			Bivariate OR (95% CI)	Conditional OR (95% CI)	Bivariate OR (95% CI)	Conditional OR (95% CI)
**Predisposing variables**						
	Female (vs male)		1.18 (0.93-1.49)	1.07 (0.83-1.37)	1.17 (0.92-1.47)	1.11 (0.83-1.49)
	Age		0.99 (0.98-0.99)*	0.99 (0.98-1.00)*	1.01 (1.00-1.02)*	1.02 (1.00-1.03)*
	**Education (vs less than high school)**					
		High school	0.46 (0.23-0.90)*	0.53 (0.26-1.08)	1.01 (0.69-1.48)	1.08 (0.72-1.62)
		Vocational school	0.71 (0.38-1.31)	0.85 (0.44-1.65)	1.09 (0.75-1.6)	1.58 (1.03-2.42)*
		College and above	0.75 (0.41-1.35)	0.94 (0.48-1.84)	1.53 (1.06-2.2)*	2.35 (1.50-3.68)*
	Currently married (vs not)		1.03 (0.82-1.30)	1.15 (0.88-1.49)	0.89 (0.70-1.13)	0.97 (0.69-1.36)
	Currently employed (vs not)		1.07 (0.84-1.35)	0.95 (0.71-1.25)	0.62 (0.49-0.80)*	0.66 (0.50-0.89)*
	**Smoking status (vs every day)**					
		Some days	1.86 (0.94-3.67)	1.6 (0.78-3.28)	0.57 (0.22-1.49)	0.54 (0.20-1.46)
		Not at all	1.19 (0.78-1.81)	1.48 (0.94-2.34)	1.14 (0.83-1.56)	0.94 (0.64-1.39)
	Family cancer history (vs not)		1.05 (0.82-1.35)	0.94 (0.72-1.23)	2.72 (2.14-3.44)*	2.23 (1.73-2.88)*
	BMI		1.00 (0.98-1.01)	0.99 (0.97-1.01)	0.98 (0.94-1.01)	0.98 (0.94-1.03)
**Enabling variables**						
	**Family annual income (vs $19,999/50,000 RMB and below)**					
		$20,000 to $49,999 (50,001 to 100,000 RMB)	0.78 (0.53-1.13)	0.81 (0.53-1.22)	0.79 (0.59-1.05)	0.70 (0.51-0.96)*
		$50,000 to $99,999 (100,001 to 150,000 RMB)	0.66 (0.45-0.94)*	0.71 (0.46-1.09)	0.72 (0.49-1.05)	0.65 (0.43-0.99)*
		$100,000 or more (150,001 RMB and above)	0.81 (0.56-1.17)	0.83 (0.52-1.34)	0.93 (0.66-1.31)	0.71 (0.48-1.06)
	**Trust in information sources**					
		Social institutions	1.02 (0.96-1.08)	1.00 (0.94-1.06)	1.01 (0.96-1.07)	.97 (0.90-1.04)
		Traditional media	1.02 (0.96-1.09)	1.01 (0.95-1.07)	1.04 (0.99-1.09)	1.01 (0.95-1.08)
		Internet	1.17 (0.99-1.38)	1.14 (0.99-1.31)	1.25 (1.06-1.47)*	1.43 (1.13-1.8)*
		Doctors	0.97 (0.79-1.19)	0.95 (0.80-1.12)	0.94 (0.82-1.07)	0.96 (0.81-1.13)
		Family and friends	0.96 (0.81-1.13)	0.97 (0.84-1.11)	0.73 (0.62-0.85)*	0.71 (0.59-0.86)*
**Need variables**						
	Perceived health status		0.77 (0.68-0.87)*	0.76 (0.65-0.87)*	0.75 (0.65-0.87)*	0.94 (0.79-1.11)
	Cancer fatalism		1.05 (1.01-1.1)*	0.99 (0.95-1.03)	1.04 (1.00-1.08)*	1.03 (0.99-1.08)
	Cancer risk		1.35 (1.19-1.54)*	1.13 (0.98-1.29)	1.25 (1.09-1.43)*	0.97 (0.82-1.16)
	Cancer fear		1.66 (1.49-1.85)*	1.59 (1.41-1.79)*	1.38 (1.24-1.54)*	1.28 (1.12-1.47)*

*Asterisks represent the coefficients that are statistically significant at the *P*=.05 level.

## Discussion

### Principal Findings

This study used HINTS 2017 of the US and HINTS-China 2017 data to compare the associations between factors related to online cancer information seeking. Under the framework of the behavioral model of health services use, the results reveal clear patterns of cross-country differences: the Chinese respondents’ internet cancer information seeking was associated more with the predisposing and enabling variables, while the US respondents’ information seeking was related more to the need variables. Specifically, the internet cancer information-seeking behavior of the Chinese respondents was associated with the predisposing characteristics educational attainment, employment status, and family cancer history, while that of the US respondents was not related to any of the predisposing characteristics. For enabling conditions, the internet cancer information seeking of the Chinese respondents was related to trust in family and friends as reliable health information sources, while that of the US respondents was not correlated with any enabling factor. For need variables, the internet cancer information seeking of the Chinese respondents was not related to perceived health status, while that of the US respondents was negatively associated with perceived health condition, and the magnitude of the association between cancer fear and internet cancer information seeking was stronger for the US respondents than for the Chinese respondents. These cross-country differences reveal that the extent to which predisposing characteristics, enabling conditions, and perceived needs are related to internet cancer information seeking is possibly subject to the nature of a country’s cultural and structural characteristics. The importance of predisposing characteristics and enabling conditions outweighs perceived needs in countries where, for example, the culture is more collectivistic or information channel credibility is of greater concern. The perceived needs of individuals may play a larger role in more individualistic cultures.

Conventional wisdom holds that people with high socioeconomic status have high internet and health literacy, which in turn allows them to use new technologies to satisfy their cognitive and emotional needs. However, the association between being employed and information seeking suggests that working overtime and having a more active social life, by-products of being employed and financially secure in China, may limit the available time for online cancer information seeking. China has a fairly strong family-oriented culture [45]. In the context of this study, as family members remain close to each other, when certain family members are diagnosed with cancer, such news spreads very quickly within the so-called networked family. Primed by such news and vividly aware of the serious psychological and physical consequences of being a cancer patient, Chinese people might go to the internet to search for cancer-related information such as preventive measures.

Additionally, the Chinese online cancer information seekers trusted family and friends as reliable information sources more than nonseekers did, which did not apply to the US respondents. This shows that when family members are trusted as sources of information, they can displace the internet as cancer information sources in China. Although Table 1 suggests that the interaction term of trust in the internet and seeking is not statistically significant, the results in Table 3 show that the conditional odds ratio of trust in the internet was positive and statistically significant only for the Chinese sample and not for the US sample. This might be attributed to the difference in the internet between China and the US. Chinese cyberspace has long been overwhelmed with misinformation about health [51]. Thus, trust in the internet may need to become a very salient factor before Chinese people decide to use the internet as a source of cancer-related information.

The US-China differences in the associations of need factors, such as perceived personal health status and cancer fear, might reveal individualistic versus collectivistic cultural influences. Perceived personal health status was associated with online cancer information seeking only for the US respondents and not for the Chinese respondents. Previous studies have suggested that people in individualistic cultures tend to pay more attention to personal wellbeing [42]. Naturally, when perceiving their health to be deteriorating, the US respondents were more likely than the Chinese respondents to take measures such as seeking information related to cancer prevention or treatment to regain psychological or physical wellbeing. The nonsignificant results for the Chinese population are consistent with a previous study analyzing HINTS-China 2012 data [27]. In that study, the authors speculated that cancer information seeking among Chinese people might be performed only for the good of others. By resorting to a comparative design, this study can offer more convincing evidence of the close relationship between culture and health information seeking. This study also found that the odds ratio of cancer fear was much smaller for the Chinese respondents than for the US respondents, which shows that cultural characteristics might influence cancer information seeking. In individualistic cultures, negative affect is considered to be an individual’s responsibility and is conceptualized as harmful, while in collectivistic cultures, negative affect is considered to be external to individuals and natural [43]. This difference sheds light on the moderating effects of culture on the correlation between negative affect (fear of cancer, in this study) and online cancer information seeking. In the context of this study, Americans’ fear of cancer might result from beliefs that cancer risks are related to unhealthy personal behaviors, so they might react to cancer fears more intensely than Chinese people and strive to correct the perceived problematic situation and regain wellbeing by seeking cancer information on the internet.

In this study, differences in the associations of age and educational attainment may be related to the composition of the sample respondents. For the Americans, the higher the age, the less likely they were to search for cancer information on the web; for the Chinese, the opposite was true. As Table 2 suggests, the US sample had a much higher average age. Commonly, younger people are more technologically savvy, and older people pay more attention to personal health. In other words, age carries two types of information here. For the US sample (older age) who already paid sufficient attention to health, age was more likely to approximate internet literacy, while for the Chinese sample (younger age) who already had sufficient internet skills, age was more likely to approximate attention to health. According to Table 2, the variation in educational attainment was lower for the Americans than for the Chinese, as almost 50% of the US respondents had a college degree and above, while the educational attainment in the Chinese sample was more equally spread across the four different categories. The lack of statistically significant results may be related to the lower variance in the predictor on the American side.

### Limitations

This study is not without limitations. First, as it tried to select the exact same set of variables to be compared, it excluded several important variables related to health cognition because either the Chinese or US survey did not measure them. Although the researchers made the utmost efforts to unify the measures of all the variables used in the analysis, some, such as trust in social institutions and information channels, were not identically designed, which may have impacted the results to some extent. This study is based on secondary data from HINTS. Explanations of cross-country/cultural differences involve speculation based on theoretical and practical reasoning, so future researchers are advised to further explore the precise mechanism of how culture influences internet cancer information seeking by measuring antecedents to variables such as cancer fear.

The study has other limitations as well. The researchers used two cross-sectional surveys, so causal relationships cannot be truly established. The data used in this study were only from the HINTS, and cross-country/cultural differences could be confirmed only with more replications based on additional sources of data. In this study, all the measures were self-reported, and in this context, cultural differences in response style are likely to occur, or respondents may differ in their interpretation of the questions.

### Conclusions

Despite these limitations, this study makes unique theoretical and practical contributions to the literature and practice. By comparing HINTS 2017 and HINTS-China 2017 data, this study found that predisposing characteristics and enabling conditions were more associated with internet cancer information seeking for the Chinese sample and that need factors were more related to information seeking for the US respondents. Such differences might reflect possible US-China differences in job environment (eg, job pressure) and culture (individualism vs collectivism and family structure). Future health communication researchers may consider incorporating cultural values into the study design when possible. Additionally, future studies in non-Western countries may consider focusing more on predisposing factors such as structural characteristics and enabling factors related to family structure. Because international charities and health nonprofits actively promote health causes in Asian and African countries, practitioners should consider not only being culturally sensitive but also placing culture at the center of their campaigns.

## Abbreviations

HINTS: Health Information National Trends Survey
